# Microbial consortia degrade several widely used organic UV filters, but a number of hydrophobic filters remain recalcitrant to biodegradation

**DOI:** 10.1007/s11356-023-31063-w

**Published:** 2023-11-27

**Authors:** Sonja K. Fagervold, Clémence Rohée, Philippe Lebaron

**Affiliations:** 1 Sorbonne Université, CNRS, Laboratoire de Biodiversité et Biotechnologies Microbiennes, LBBM, Observatoire Océanologique, 66650 Banyuls-sur-mer, France; 2 Pierre Fabre Dermo-Cosmétique et Personal Care, Centre de Recherche & Développement Pierre Fabre, 31000 Toulouse, France

**Keywords:** Organic UV filters, Environmental fate, Biodegradation, Consortia, Wastewater sludge, Pure bacterial strains

## Abstract

Organic UV filters are important ingredients in many personal care products, including sunscreens. Evaluating the biodegradability of organic UV filters is key to estimate their recalcitrance and environmental fate and thus central to their overall environmental risk assessment. In order to further understand the degradation process, the aim was to investigate whether specific consortia could degrade certain UV filters. Several bacterial strains were isolated from enrichment cultures actively degrading octocrylene (OC), butyl methoxydibenzoylmethane (BM), homosalate (HS), and 2-ethylhexyl salicylate (ES) and were utilized to construct an in-house consortium. This synthetic consortium contained 27 bacterial strains and degraded OC, BM, HS, and ES 60–80% after 12 days, but not benzophenone-3 (BP3), methoxyphenyl triazine (BEMT), methylene bis-benzotriazolyl tetramethylbutylphenol (MBBT), diethylhexyl butamido triazone (DBT), ethylhexyl triazone (EHT), or diethylamino hydroxybenzoyl hexyl benzoate (DHHB). Furthermore, several commercial microbial mixtures from Greencell were tested to assess their degradation activity toward the same organic UV filters. ES and HS were degraded by some of the commercial consortia, but to a lesser extent. The rest of the tested UV filters were not degraded by any of the commercial bacterial mixes. These results confirm that some organic UV filters are recalcitrant to biodegradation, while others are degraded by a specific set of microorganisms.

**Supplementary Information:**

The online version contains supplementary material available at 10.1007/s11356-023-31063-w.

## Introduction

UV filters are important ingredients in many personal care products (PCPs), including sunscreens, and play a critical role in protecting humans against potentially harmful UV rays. Commercial sunscreen formulations are complex mixtures with organic UV filters often being a key ingredient, sometimes reaching a high percentage of the final product (Osterwalder et al. 2014). Due to the relatively high volume of use, there are concerns regarding the potential toxic effects of UV filters on humans as well as the ecotoxicological effects that UV filters may have on wildlife. As to human exposure and effects, there is concern regarding the penetration of the organic UV filters through the skin barrier and the uptake of these compounds in the body. Indeed, some organic UV filters have been found in breast milk (Hany and Nagel 1995; Schlumpf et al. 2010) and the urine of children (Lu et al. 2018). Additional potential effects are, among others, allergic reactions, cytotoxicity, and estrogenic effects (reviewed by Gilbert et al. (2013) and Egambaram et al. (2020)). With regard to ecotoxicological effects, some of the most problematic are the effects on corals (Downs et al. 2014; Fel et al. 2019; He et al. 2019) and other marine wildlife (recently reviewed by Lozano et al. (2020a), as well as the potential for bioaccumulation (Gago-Ferrero et al. 2015; Alonso et al. 2015; Molins-Delgado et al. 2017; Díaz-Cruz et al. 2019), due to the relative lipophilic nature of many of the organic UV filters.

Inorganic UV filters, such as zinc oxide and titanium dioxide, protect against UV rays by reflecting and scattering incoming rays, while organic UV filters function by absorbing the energy in UV rays through conformational changes in the chemical structure (Chisvert and Salvador 2007). Although there are 26 different organic UV filters currently allowed in cosmetic products (EU 2021), the focus here is on a subset of 10 organic UV filters with different structures and solubilities, several of which have been branded as “new generation,” “reef safe,” and/or “eco friendly” (Miller et al. 2021; Varrella et al. 2022). A common feature of organic UV filters is that they contain aromatic structures, something that may also render them less biodegradable. Indeed, relatively few organic UV filters have been shown to be degraded in ex situ experiments, and there are even fewer reports where the microorganism responsible for the degradation is identified. Benzophenone-3 (BP3) has been reported to be degraded in several studies involving yeast and WWTP sludge (Fujii and Kikuchi 2005), *Trametes versicolor* and sterilized WWTP sludge (Badia-Fabregat et al. 2012), WWTP sludge microcosms (Liu et al. 2013; Fagervold et al. 2021), and aqueous and sediment microcosms (Liu et al. 2013). Furthermore, several microorganisms have been identified as BP3 degraders, namely, *Sphingomonas wittichii* strain BP14P (Fagervold et al. 2021), *Methylophilus* sp. strain FP-6 (Jin et al. 2019), and *Rhodococcus oxybenzonivorans* sp. nov. (Baek et al. 2022a, b). For the latter, the enzymes in the biodegradation pathway have been elucidated for the type strain *Rhodococcus oxybenzonivorans* sp. S2-17 (Baek et al. 2022b), and a degradation pathway has been proposed. Octocrylene (OC) was also degraded by *Trametes versicolor* with sterilized WWTP sludge (Badia-Fabregat et al. 2012), by aquifer materials and by WWTP sludge microcosms (Suleiman et al. 2019; Fagervold and Lebaron 2022). In addition, several bacterial strains have been identified as capable of OC degradation, namely, *Gordonia* sp. strain OC_S5 and *Sphingopyxis* sp. strain OC_4D (Fagervold and Lebaron 2022). 2-Ethylhexyl salicylate (ES), homosalate (HS), and butyl methoxydibenzoylmethane (BM) have also been shown to degrade in WWTP sludge microcosms (Fagervold and Lebaron 2022), but the bacterial strains responsible for this degradation were not identified. These were initially non-enriched sludge microcosms. Several organic UV filters have been shown to be recalcitrant to biodegradation in non-enriched sludge microcosm experiments (Fagervold and Lebaron 2022), including methoxyphenyl triazine (BEMT), methylene bis-benzotriazolyl tetramethylbutylphenol (MBBT), and diethylhexyl butamido triazone (DBT). Furthermore, ethylhexyl triazone (EHT) and diethylamino hydroxybenzoyl hexyl benzoate (DHHB) have not yet been tested for degradation in microcosms. Thus, in summary, of the 10 organic UV filters targeted here, 5 UV filters have been shown to be degradable (BP3, HS, OC, ES and BM), and 5 have either not been tested or been shown to be recalcitrant (EHT, DHHB, BEMT, MBBT, and DBT).

It has long been established that microorganisms often work together to degrade certain organic compounds and pollutants (see Bhatt et al. (2021) and Zhang and Zhang (2022) for recent reviews). Indeed, it is often the case that the performance of a consortium of microorganisms is better than that of single strains. Furthermore, a simplified microbial consortium can be constructed without losing the function or efficiency of the process of interest (Kang et al. 2020; Liang et al. 2022). Indeed, enrichment cultures that have already undergone over 20 transfers and are still able to efficiently degrade specific organic UV filters are the basis for the current work (Fagervold and Lebaron 2022). These consortia were enriched by a “top-down” strategy (Liang et al. 2022), and the microbial communities in each of the enrichment cultures were specific for each different UV filter added (Fagervold and Lebaron 2022).

Here, the goal was to investigate whether synthetic consortia of microorganisms are capable of degrading a more expanded list of organic UV filters than previously known, hopefully furthering the understanding of possible hurdles in biodegradation process. The hypothesis was that increased concentrations of microorganisms with degradation capabilities would lead to degradation of recalcitrant UV filters. Furthermore, the effect of adding the degradable UV filter to cultures with recalcitrant filters was tested with the hypothesis that this would have a stimulating effect on the biodegradation of recalcitrant filters. This aim led us to expand on previous work and increased the repertoire of bacterial strains capable of degrading organic UV filters. Thus, several strains degrading ES, HS, OC, and BM were isolated from previously characterized enrichment cultures that actively degraded ES, HS, OC, and BM (Fagervold and Lebaron 2022). The isolated strains were then utilized to create an in-house consortium. This in-house consortium, as well as several commercially available consortia, was tested for biodegradation activity toward both “degradable” UV filters (BP3, HS, OC, ES, and BM) and “recalcitrant” UV filters (EHT, DHHB, BEMT, MBBT, and DBT).

## Materials and methods

### Chemicals

Pierre Fabre Dermo-cosmetic (France) provided some of the UV filters used for biodegradation studies, including ES, HS, BM, OC, BEMT, and DBT. MBBT was purchased from Sigma-Aldrich (Saint-Quentin-Fallavier, France). Analytical standards for the UV filters mentioned above were purchased from Sigma-Aldrich, as were analytical-grade dichloromethane (DCM), methanol, and formic acid (98%). Pure water was obtained from an Elga Purelab Flex System (Veolia LabWater STI, Antony, France). Glassware was cleaned with DCM and calcinated at 450 °C for 2 h to remove traces of organic matter.

### Culture methods and isolation of strains

WWTP enrichment cultures degrading OC, BM, ES, and HS were used as a source to isolate microorganisms involved in organic UV filter degradation processes. These enrichment cultures have been previously characterized (Fagervold et al. 2021; Fagervold and Lebaron 2022). Briefly, each culture contained 2 g of inert sand, 50 mL of minimal media (OECD 301), and an individual UV filter at a concentration of 100 µg/mL. The UV filters were added by first dissolving the selected UV filter in acetone and then adding the acetone to the Erlenmeyer flasks (100 mL) containing the inert sand. The acetone was allowed to evaporate before the addition of media and subsequent autoclaving. After inoculation, the Erlenmeyer flasks were incubated at 25 °C in the dark on a rotary shaker at 100 rpm after inoculation. These enrichment cultures were transferred over 20 times over several years with only one UV filter available as a carbon source before being used as a source for isolation of putative degrading strains.

**Fig. 1:**
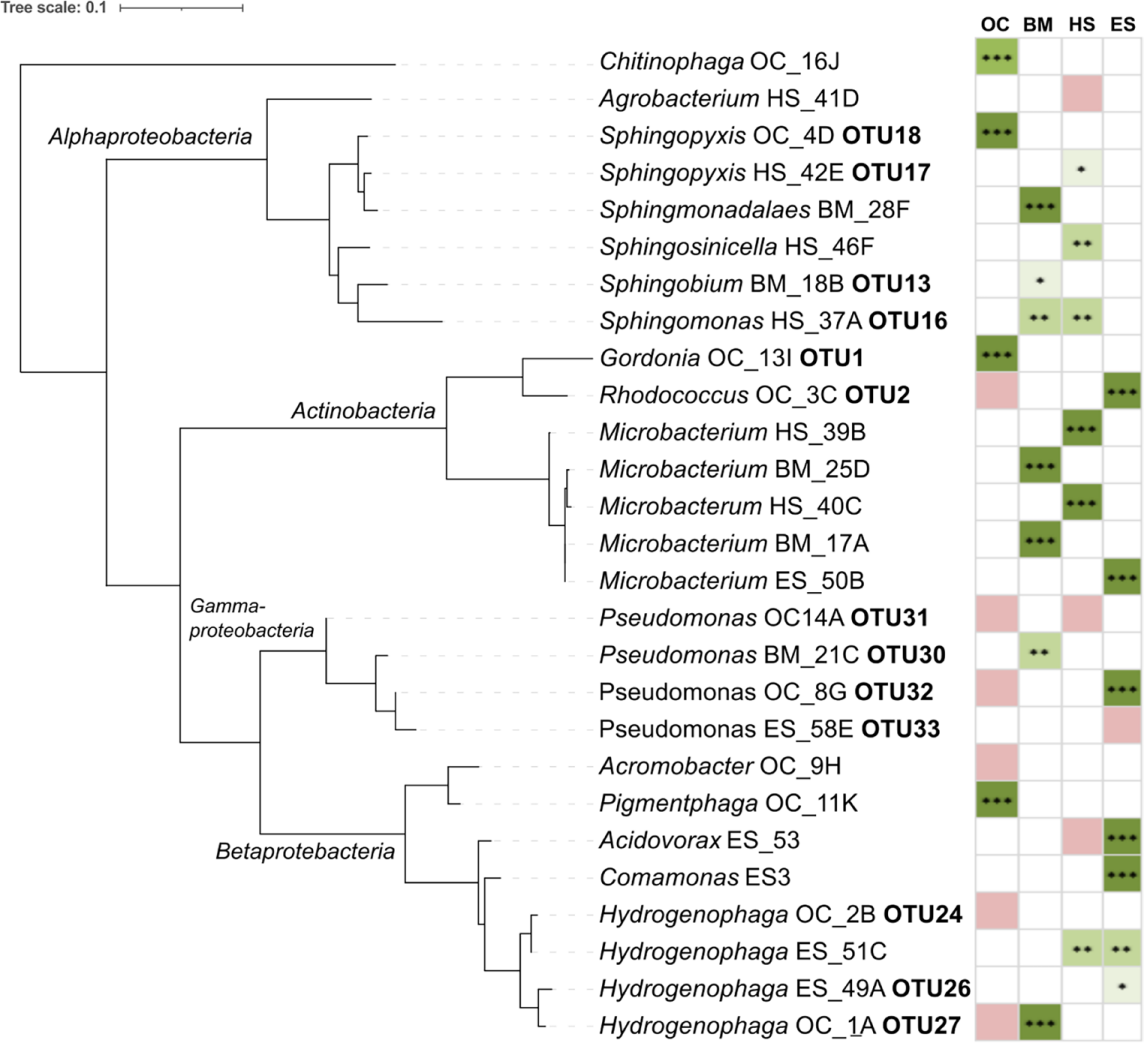
Maximum likelihood phylogenetic tree of isolated strains and their degradation capability (right). Green and three stars represent strains that degraded the selected UV filter over 70% after 20 days, light green and two stars represents between 30 and 70% degradation, pale green and one star represents between 10 and 29% degradation, and red represents strains that degraded the targeted UV filter less than 10% after 30 days

Isolation of putative degrading bacteria from the enrichments was performed as described earlier for BP3-degrading bacteria and OC-degrading bacteria (Fagervold et al. 2021; Fagervold and Lebaron 2022). Briefly, 1 mL of supernatant from enrichment cultures was harvested, and this supernatant was diluted 1000-fold before spreading 100 µL on R2A agar (Sigma-Aldrich) plates. The plates were incubated in the dark at 25 °C for 2 weeks. Colonies with distinct morphology were picked and serially passaged on agar plates until achieving purity. Screening of bacterial isolates was performed by Automated Ribosomal Intergenic Spacer Analysis (ARISA) and colony description. Only those that were different were subsequently sequenced. For conservation, bacterial isolates were grown in R2A Broth (Acumedia, Neogen Culture Media) before the addition (1:1 vol/vol) of glycerol (70% v/v) and 5% dimethyl sulfoxide. The cells were stored at − 80 °C and added to the Banyuls Bacterial Culture Collection (https://banyuls-bacterial-culture-collection.fr/). Sequences of the unique strains depicted in Fig. 1 have been submitted to GenBank under accession numbers OP985055-OP985077, except for *Gordonia* OC_13I, which is 100% identical to *Gordonia* sp. *strain* OC_5C (OL457617); *Sphingopyxis* OC_4D, which has been published previously (OL457616); *Pseudomonas* OC14A, which is 100% identical to *Pseudomonas* sp. *strain* OC_S1 (OL457619); and Hydrogenophaga OC_2B, which is 100% identical to *Hydrogenophaga* sp. strain OC_S4 (OL457618).

### Preparation of in-house consortium and Greencell microbial mixes

For the in-house consortium, the goal was to make a mix of strains where each strain was present in equal amounts, approximately 2 × 10^6^ cells per mL of each strain. The different strains were grown in R2A broth for 24 to 48 h to an optical density (OD 600 nm) of 0.4 to 1.4, depending on the strain, and accurate quantification was then performed by flow cytometry, as described earlier (Fagervold et al. 2021). Based upon the numbers of cells per milliliter attained from flow cytometry, a mix of the different strains was made, and this mixture was added to the tubes for the biodegradation assay, resulting in a theoretical final concentration of 2 × 10^6^ cells per mL of each strain.

Five different microbial mixes were received from Greencell. Four of these mixtures were in powder form, and one was in liquid form. Details regarding commercial names, the identity of the microorganism, and the minimal quantity in the mixes are presented in Table 1. These consortia have been specifically developed for various environmental applications, such as grease tank treatment (MycoEpur-BG), polluted soil treatment (MycoEpur-TP), improving composting (MycoEpur-CP), and wastewater treatment plant performance (MycoEpur-P). Thus, in contrast to the in-house consortium, these consortia are not specifically adapted to the degradation of organic UV filters. Upon delivery, the Greencell microbial mixtures were stored at 4 °C until the start of the experiments. Approximately 2–3 g of the powder was dissolved in 10 mL of Milli-Q water and mixed by rigorous shaking. Then, 0.3 mL of this slurry was used for the degradation assays.

**Table 1:** Commercial consortia names and descriptions

Nr	Greencell name	Description	Quantity
Mix 1	MycoEpur-BG “Type 82”	3 bacterial strains (2 *Bacillus* sp. and 1 *Pseudomonas* sp.)	> 10^8^ CFU/g
Mix 2	MycoEpur-TP “Type 87”	2 bacterial strains (*Pseudomonas* sp. and *Rhodococcus* sp.*)* and a fungal *Phanerochaete* sp. strain	> 1 × 10^7^ CFU/g bacteria and > 1 × 10^4^ CFU/g fungi
Mix 3	CM-DEV_OBS	*Micrococcus* sp., *Bacillus* sp. (2 strains), *Pseudomonas* sp. and *Rhodococcus* sp.	
Mix 4	MycoEpur-CP “Neutraliere”	2 *Bacillus* sp. strains and 3 filamentous fungi strains (*Mucor* sp.*, A*sp*ergillus* sp. and *Galactomyces* sp.)	> 3 × 10^6^ CFU/g bacteria and > 1.7 × 10^4^ CFU/g fungi
Mix 5	MycoEpur-P “Type 75”	2 *Pseudomonas* sp. strains 3 filamentous fungi strains (2 *Trichoderma* sp. strains and *Phanerochaete* sp.)	> 1 × 10^7^ CFU/mL bacteria and > 1 × 10^3^ CFU/mL fungi

### Biodegradation assays

Each of the different isolates was tested for their degradation capability dependent on the enrichment culture from which the isolate was derived. For example, the isolates from the enrichment culture degrading OC were tested for OC degradation. Both minimal media and minimal media with the addition of R2A broth (R2B) (20%) were used for these assays to investigate whether the addition of more nutrients (R2B) would have an influence on degradation. Furthermore, in addition to each of the isolates, both positive and negative controls were applied, namely: (i) sterile controls to control for abiotic degradation; (ii) “enrichment culture”; in which the original enrichment cultures were tested for degradation; and (iii) a mix of all the different isolates (for example, for OC, all the isolates tested for OC degradation were also tested as a mix). Biodegradation assays were performed as described previously (Fagervold et al. 2021; Fagervold and Lebaron 2022) in 15-mL glass tubes with Teflon-lined caps (Pyrex, Analytic lab, France). Each tube contained 0.2 g of inert sand, 3 mL of minimal freshwater media or minimal media with 20% R2B, and the target UV filter at a concentration of approximately 100 µg/mL. The UV filters were added as described above, by dissolving the filter in acetone, followed by evaporation in the tubes before media were added. The tubes were then autoclaved. For the biodegradation assays of single strains, 150-µL washed cell suspension was used as inoculate. The tubes were incubated at 25 °C in the dark on a rotary shaker at 100 rpm, and triplicate tubes were sacrificed for extractions at each time point.

For the biodegradation assays with consortia as inoculum, the medium used was exclusively minimal medium with 20% R2A broth. The target UV filters were BP3, HS, ES, OC, BM, BEMT, MBBT, DBT, EHT, and DHHB. These target UV filters were tested individually with the in-house consortium. In addition, the UV filters deemed “recalcitrant,” namely, BEMT, MBBT, DBT, EHT, and DHHB, were also tested with a mix of more “easily degradable UV filters” (BP3, HS, ES, OC, BM) at a lower concentration (approximately 10 µg/mL). The biodegradation assay for the Greencell microbial mixes was performed with a mix of “easily degradable UV filters” (BP3, HS, ES, OC, BM) and recalcitrant UV filters (BEMT, MBBT, DBT, EHT, and DHHB) at an approximate final concentration of 50 µg/mL of each filter.

### UV filter extractions and HPLC analysis

Extractions of organic UV filters for the biodegradation assay were performed as previously described (Fagervold et al. 2021; Fagervold and Lebaron 2022). Briefly, whole tubes were extracted directly in DCM, shaken overnight and injected into an Ultimate 3000TM HPLC system equipped with a DAD detector (Thermo Fisher Scientific). The injection volume was 5 µL, and a Phenomenex Kinetex Biphenyl column with 2.6 µm particle size and dimensions 150 × 4.6 mm was used. The data acquisition software was Chromeleon™ 7.2 (Thermo Fisher Scientific). Calibration curves and retention times of all the different UV filters were determined as described previously (Fagervold et al. 2019).

### Molecular biology methods

DNA from single bacterial colonies used for screening was recovered using a rapid lysing technique. Single colonies were placed into 50 µL of Milli-Q water and subjected to three cycles of rapid freezing in liquid N_2_ followed by rapid thawing at 70 °C. The resulting lysate was used for screening using Automated Ribosomal Intergenic Spacer Analysis (ARISA). DNA from consortia and isolates (for 16S rRNA sequencing) was extracted using the Wizard Genomic DNA purification Kit (Promega, Charbonnières-les-Bains, France) following the manufacturer’s instructions. Briefly, 2 mL of culture (either isolate or consortium) was centrifuged at 14,500 × g for 10 min in a microcentrifuge tube. The pellet was resuspended in 300 µL of Milli-Q water before adding 600 µL of Nuclei Lysing Solution. After purification and drying, the DNA was resuspended in 100 µL of rehydration solution.

ARISA of isolate lysates was performed as described previously (Fisher and Triplett 1999) with modifications (Fagervold et al. 2021). Briefly, the intergenic spacer primers 1406F and 23SRY (Fisher and Triplett 1999) were utilized for the initial PCR. Then, a 16-capillary Applied Biosystems Sequencer 3130XL (Thermo Fisher Scientific) together with the internal standard MapMarker® X-Rhodamine Labeled 50–1000 bp (Bioventures Inc., TN, USA) were used for the determination of peak lengths. These peak lengths were the basis for determining whether the isolates were unique.

Dideoxy reaction Sanger sequencing was performed on strains deemed unique. This procedure has been described previously (Fagervold et al. 2021). Briefly, universal bacterial primers 27F and 1492R were used in the first PCR, followed by the internal primers 907R, 804F, and S8 for the sequencing reactions. After purification, the BigDye™ Terminator v3.1 Cycle Sequencing Kit (Thermo Fischer) was used following the manufacturer’s protocol and run on a 16-capillary Applied Biosystems Sequencer 3130XL.

### Phylogenetic, statistical and pathway prediction analysis

For the phylogenetic tree, sequences were aligned using the Silva aligner (https://www.arb-silva.de/aligner/). The alignment was then curated with Gblocks, and a total of 688 positions were used for construction of a maximum likelihood phylogenetic tree (PhyML) using the default substitution model and online tools (http://www.phylogeny.fr) (Dereeper et al. 2008). The resulting tree was visualized using iTOL (Letunic and Bork 2021). For the Greencell biodegradation assays, the results from sterile controls (SCs) at day 30 and the live cultures at day 30 were tested for significant differences (*p* < 0.01) using Student’s *t* test (unpaired). Possible microbial-mediated degradation pathways can be predicted by an online tool (http://eawag-bbd.ethz.ch/predict/) (Gao et al. 2010). This tool was used to investigate the probable first steps in the degradation pathways of the different UV filters. However, full degradation pathway determination is outside the scope of this study.

## Results

### Isolation of microorganisms from enrichment cultures and their degradation capacities

WWTP enrichment cultures degrading ES, HS, BM, and OC (Fagervold and Lebaron 2022) were used as a source for isolating microorganisms involved in organic UV filter degradation processes. Twenty isolates were screened from each actively degrading enrichment culture (ES, HS, BM, and OC) by ARISA. This resulted in 7 to 10 different strains (Table 2) from each of the enrichment cultures. Most of the strains were closely related to already-described species with 16S rRNA gene sequence identities from 97.22 to 99.92% (Table 3). Furthermore, many, but not all, strains could be traced back to the enrichment cultures as “OTUs,” shown to be present by Illumina sequencing (Fagervold and Lebaron 2022).

**Table 2:** Screening of isolates

UV filter	Nr. isolates screened	Nr. different strains	Degradation^a^
OC	20	10	4
BM	20	7	6
HS	20	9	5
ES	20	8	6

^a^Number of strains with a minimal degradation activity of 30% in minimal media or with 20% r2B after 20–30 days

**Table 3:** Characterization of the different isolates

Isolate^a^	Colony^b^	OTU^c^	Closest described strain (accession number)	Ident ^d^
OC_1A	Cream translucent	OTU 27	*Hydrogenophaga intermedia* strain S1 (NR_024856)	98.98
OC_2B	Light yellow diffuse	OTU 24	*Hydrogenophaga electricum* strain AR20 (NR_132676)	99.73
OC_3C	Peach	OTU 2	*Rhodococcus qingshengii* strain djl-6–2 (NR_115708)	99.80
OC_4D	Yellow	OTU 18	*Sphingopyxis terrae* subsp. *ummariensis* strain UI2 (NR_116018)	99.79
OC_8G	Yellow diffuse	OTU 32	*Pseudomonas pseudoalcaligenes* strain Stanier 63 (xx)	99.05
OC_9H	Cream glistening		*Achromobacter pulmonis* strain R-16442 (NR_117644)	99.93
OC_13I	Pink	OTU 1	*Gordonia alkanivorans* strain HKI 0136 (NR_026488)	99.80
OC_16J	Yellow diffuse		*Chitinophaga arvensicola* strain NBRC 14973 (NR_113715)	97.31
OC_11K	Cream/pink		*Pigmentiphaga kullae* strain K24 (NR_025112)	98.84
OC14A	Cream glistening	OTU 31	*Pseudomonas delhiensis* strain RLD-1(NR_043731)	99.44
BM_17A	Light yellow		*Microbacterium phyllosphaerae* strain P 369/06 (NR_025405)	98.84
BM_18B	Small yellow	OTU 13	*Sphingobium phenoxybenzoativorans* strain SC_3 (NR_135895)	99.78
BM_21C	Light yellow diffuse	OTU 30	*Pseudomonas aeruginosa* strain DSM 50071	99.80
BM_25D	Light yellow		*Microbacterium schleiferi* strain DSM 20489	99.59
BM_27E	Cream	OTU 27	*Hydrogenophaga intermedia* strain S1 (NR_024856)	99.04
BM_28F	White		*Sphingopyxis apnaciterrulae* strain DCY34 (NR_116164)	98.32
BM_29G	Cream/peach	OTU 16	*Sphingomonas wittichii* RW1 (NR_074268.1)	99.86
HS_37A	Cream/peach	OTU 16	*Sphingomonas wittichii* DC-6 (KC410868)	99.93
HS_39B	Peach glistening		*Microbacterium dextranolyticum* strain DSM 8607 (NR_044934)	98.70
HS_40C	Small translucent		*Microbacterium invictum* strain DC-200 (NR_042708)	98.84
HS_41D	Light yellow		*Beijerinckia fluminensis* strain UQM 1685 (NR_116306)	99.93
HS_42E	Light yellow	OTU 17	*Sphingopyxis taejonensis* strain JSS-54 (NR_024999)	98.79
HS_46F	Peach		*Sphingosinicella microcystinivorans* strain Y2 (NR_040927)	98.87
HS_65G	Cream		*Acidovorax wautersii* strain NF 1078 (NR_118410)	99.18
HS_67H	Yellow diffuse		*Hydrogenophaga temperata* strain TR7-01 (NR_132598)	99.45
HS_S1	Cream glistening		P*seudomonas delhiensis* strain RLD-1 (NR_043731)	99.44
ES_49A	Translucent	OTU 26	*Hydrogenophaga defluvii* strain BSB (NR_029024)	99.39
ES_50B	Cream		*Microbacterium paraoxydans* (NR_025548)	98.58
ES_51C	Yellow diffuse		*Hydrogenophaga temperata* strain TR7-01 (NR_132598)	99.45
ES_54D	Yellow	OTU 32	*Pseudomonas chengduensis* strain MBR (NR_125523)	98.59
ES_58E	Peach	OTU 33	*Pseudomonas putida* strain ICMP 2758 (NR_114794)	99.70
ES_64F	Cream	OTU 2	*Rhodococcus qingshengii* strain djl-6–2 (NR_115708)	99.78
ES_53	Cream		*Acidovorax wautersii* strain NF 1078 (NR_118410)	99.53
ES_S3	Cream		*Comamonas terrigena* strain NBRC 13299 (NR_113613)	97.22

^a^ = isolates with prefix “OC” were isolated from OC degrading enrichment cultures, the same for “BM”, “HS” and “ES”

^b^ = Color and other distinguishing feature of colonies

^c^ = the corresponding OTU from enrichment cultures described previously (Fagervold, 2022)

^d^ = Identity to the nearest top hit using Basic Local Alignment Search Tool (BLAST) search of described species (rRNA/ITS databases)

The degradation capacities of the different isolates were tested, and the results are presented in Figures S1, S2, S3 and S4. Enrichment cultures and the mix of isolates served as positive controls and showed clear degradation of all four UV filters, while the sterile control showed no significant degradation. Regarding the degradation capability of the specific strains, the results are summarized in Fig. 1. However, in instances where two isolates isolated from different enrichment cultures were 100% identical, only one strain is depicted in Fig. 1. For example, strain “Hydrogenophaga OC_1A” represents isolate OC_1A (Table 3; Figure S1) and BM_27E from BM-degrading cultures (Table 3; Figure S2).

Of the 10 different isolates tested for OC degradation, 4 clearly showed degradation activity, namely, stains OC_4D (*Sphingopyxis*), OC_13I (*Gordonia*), OC_11K (*Pigmentiphaga*), and OC_16J (*Chitinophaga*). The first three isolates exhibited clear degradation in minimal media with or without the addition of R2B, while strain OC_16J only degraded with minimal media, not when grown on more rich media. Furthermore, strain *Sphingopyxis* OC_4D and strain *Gordonia* OC_13I have previously been identified as OC degraders (Fagervold and Lebaron 2022). Previous results were ambiguous regarding the capability of a *Hydrogenophaga* strain to degrade OC (Fagervold and Lebaron 2022). Here, we show that the two *Hydrogenophaga* strains tested did not degrade OC.

All of the strains tested were capable of degrading BM to some extent (Figure S2); however, for strain BM_18B (*Sphingobium*), the degradation was minimal, with only 15 and 25% degradation in minimal media and minimal + R2B media, respectively, after 30 days of incubation. The same was not true for HS, where only two isolates, HS_39B (*Microbacterium*) and HS_40 (*Microbacterium*), clearly degraded HS after 20 days in both minimal media and minimal media with R2B. Strain HS_46F (*Sphingosinicella*) degraded HS to some extent in the presence of other carbon sources, as did strain HS_67H (*Hydrogenophaga*, ES51_C in Fig. 1) and strain HS_42E (*Sphingopyxis*.)

Most of the strains tested for ES degradation exhibited a clear degradation capacity, with over 80% degradation after 20 days, as was the case for strains ES_50B (*Microbacterium*), ES_54D (*Pseudomonas*), ES_64F (*Rhodococcus*), ES_53 (*Comamonas*), and ES_S3 (*Acidovorax*), while ES_51C (*Hydrogenophaga*) degraded 61% of the ES present after 20 days. In addition, strain ES_49A (*Hydrogenophaga*) degraded ES to a much lesser degree, with 28% degradation after 20 days.

### Biodegradation assays with synthetic bacterial consortia

**Fig. 2:**
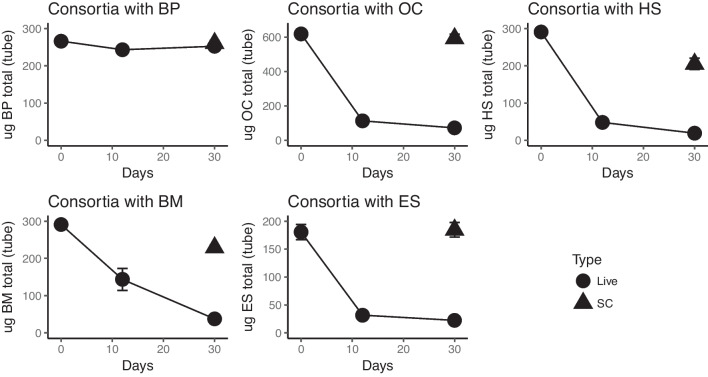
Biodegradation assays of the “degradable” UV filters with the synthetic bacterial consortia. UV filter concentrations (*y*-axis) over time (*x*-axis) in live cultures with active consortia (circles) and sterile controls without added consortia (triangles)

**Fig. 3:**
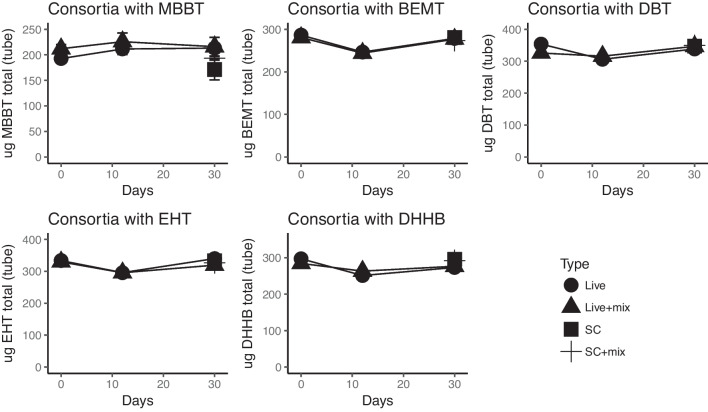
Biodegradation assays of the “recalcitrant” UV filters with the synthetic bacterial consortia. UV filter concentrations (*y*-axis) over time (*x*-axis) in live cultures with active consortia (circles), in live culture with the addition of the “easily degradable” UV filter mix (triangles), sterile controls without added consortia (squares) and sterile controls with added mix of UV filters (cross)

The in-house consortium contained 27 different strains (shown in Fig. 1) from different phylogenetic groups. This consortium was tested for degradation capabilities towards all 10 organic UV filters separately. The consortia clearly degraded OC, HS, ES, and BM after just 12 days of incubation (Fig. 2). Surprisingly, BP3 was not degraded, even after 30 days. Furthermore, in an attempt to stimulate the degradation of the recalcitrant filters, a mix of “easily degradable” UV filters was added to tubes containing each of the recalcitrant UV filters. However, this did not lead to any degradation of the recalcitrant filters (Fig. 3). Thus, the addition of the degradable filters had no observed stimulating affect.

**Fig. 4:**
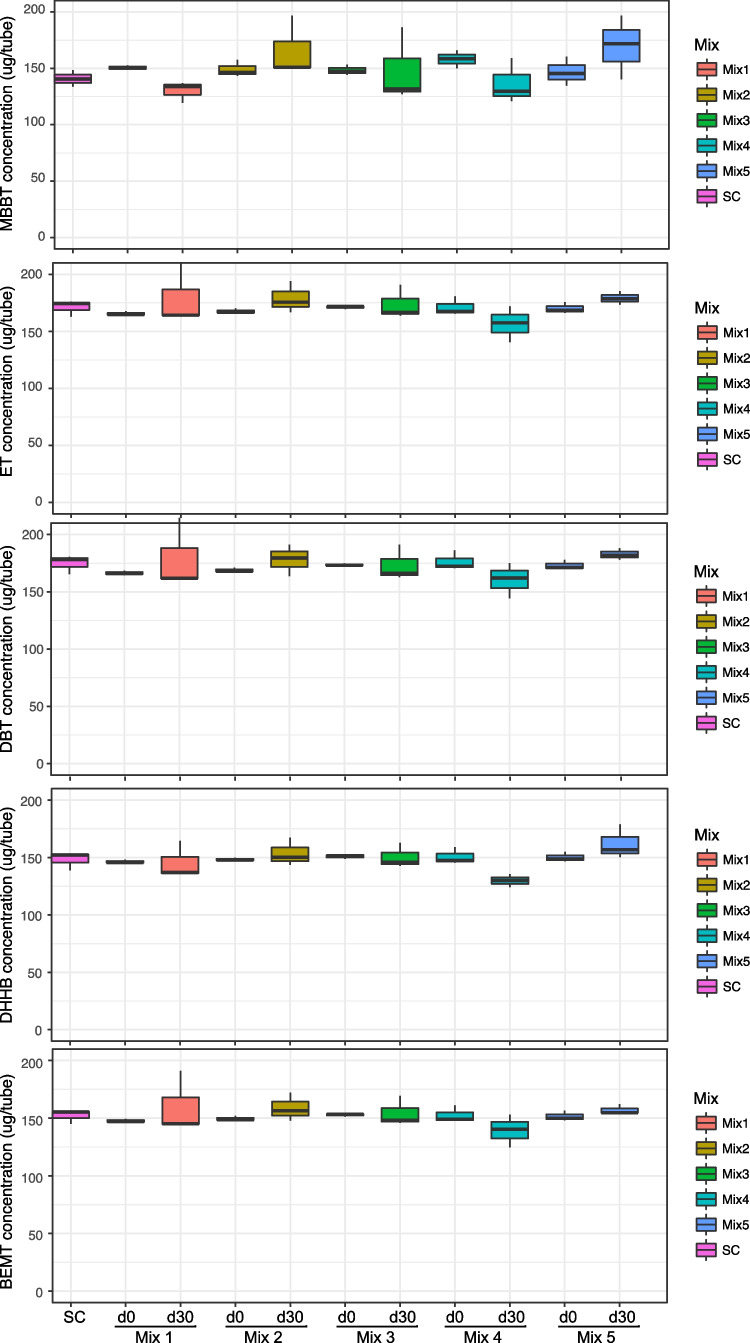
Boxplots showing the concentrations of different “recalcitrant” UV filters in SC (day 30), at day 0 and day 30 in live cultures with the different Greencell microbial mixes

**Fig. 5:**
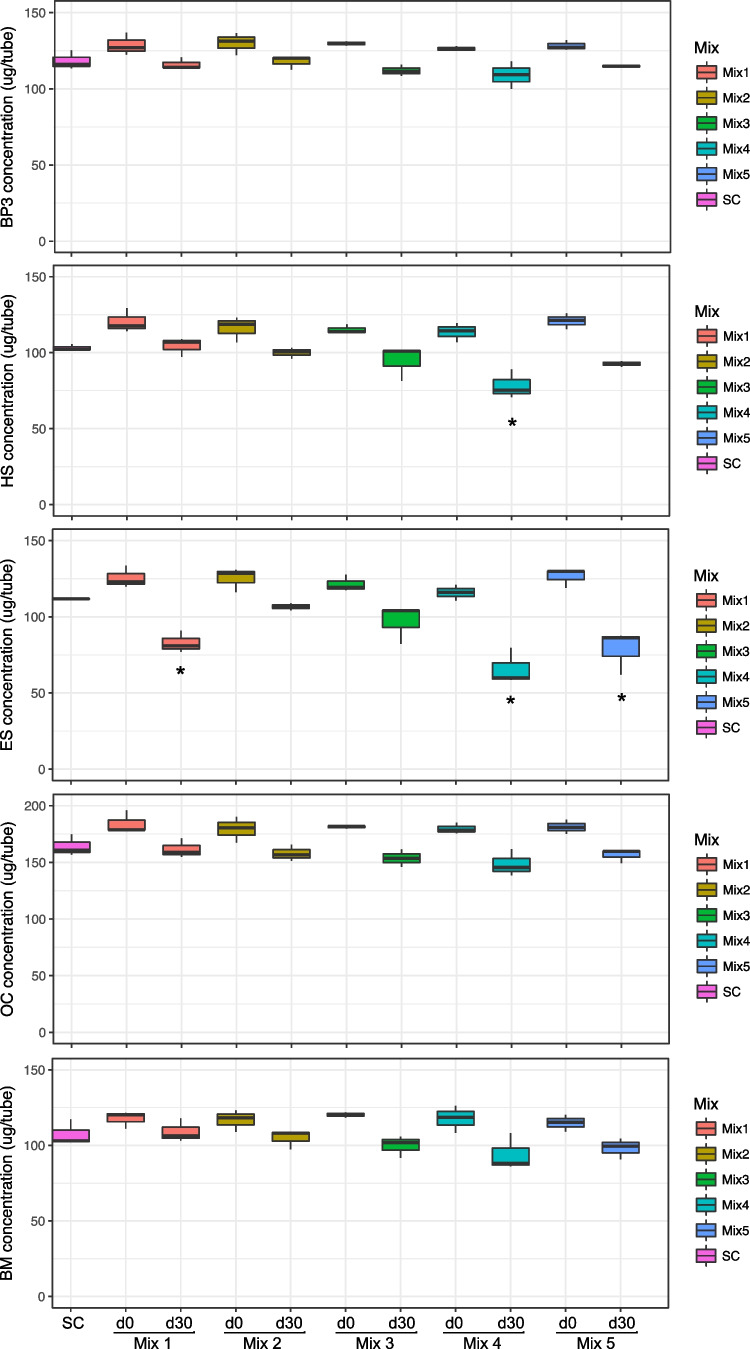
Boxplots showing the concentrations of different “degradable” UV filters in SC (day 30), at day 0 and day 30 in live cultures with the different Greencell microbial mixes. Asterisk = The result is significant at p < .01

Degradation assays of the five consortia from Greencell were carried out with a mix of “easily degradable” UV filters tested together, and a mix of “recalcitrant” UV filters tested together. Figure 4 shows the “recalcitrant” UV filters at day 0 and day 30 for the live cultures and in SC day 30 samples. To investigate whether any degradation occurred after 30 days of incubation, one should compare the results with the SC, which was also taken at day 30. None of the “recalcitrant” filters was degraded by any of the Greencell bacterial mixes (Fig. 4). However, some of the Greencell bacterial mixes had some activity toward some of the “easily” degradable UV filters (Fig. 5). Mix 4 degraded HS by 24% and ES by 40% after 30 days. In addition, ES was also degraded by mix 1 (25%) and mix 5 (29%). However, none of the Greencell mixes exhibited degradation activity toward BP, OC, or BM.

### Possible degradation pathways of the different UV filters.

The possible degradation pathways for the filters BP, BM, OC, ES, and HS, predicted by an online tool (http://eawag-bbd.ethz.ch/predict/) (Gao et al. 2010), are presented in “supplemental text 1” and Figures S5, S6, S7, and S8. The recalcitrant filters were not elucidated in the same way, but the predicted first reactions are shown in Table 4. Many of the predicted pathways start with monooxygenase activities as well as esterase/hydrolase activities. Thus, the predicted enzymes needed for degradation of both the “degradable” and “recalcitrant’ are similar in many cases.

**Table 4:**
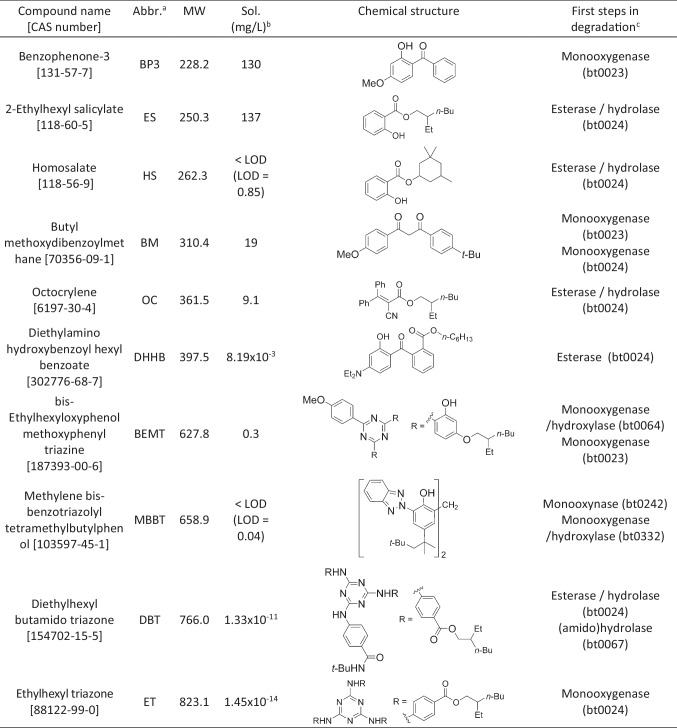
Chemical characteristics and structures of the targeted organic UV filters

^a^Abbreviation

^b^Freshwater solubility at 25 °C (mg/L). Experimental data. See protocol in Fagervold et al. (2019). LOD, limit of detection. Except for DHHB, ET and DBT which is taken fromRamos et al. (2016)

^c^As predicted by the be predicted by an online tool (http://eawag-bbd.ethz.ch/predict/)

## Discussion

### Phylogenetic groups isolated from enrichment cultures and their possible roles

Twenty-seven different strains were isolated from actively degrading enrichment cultures and subsequently used to build the in-house synthetic consortium. These strains belonged to different taxonomic groups: seven strains belonged to *Actinobacteria*, seven to *Alphaproteobacteria*, eight to *Betaprotebacteria*, and four to *Gammaproteobacteria*, all of which fell within the same genus (*Pseudomonas*), and one strain belonged to the phylum *Bacteroidetes* (genus *Chitinophaga*)(Fig. 1). Of the 27 strains, 19 exhibited clear degradation capacities (> 30%, two stars or more in Fig. 1) of OC, BM, HS, or ES.

The *actinobacterial* strains all showed high degradation capacities toward UV filters. Indeed*, Actinobacteria* are known to degrade a wide variety of organic molecules. Two of the *actinobacterial* strains were detected of in enrichment cultures, by Illumina sequencing namely, OTU 1 and OTU 2. *Gordonia* sp. strain OC_13I OTU1 is 100% identical to the previously isolated *Gordonia* sp. strain OC_S5 (Fagervold and Lebaron 2022). *Rhodococcus* sp. OC_3C (same as ES_64F) OTU2 readily degraded ES but not OC. This strain is very close (Table 3) to a strain isolated from soil that could degrade the benzimidazole fungicide carbendazim (Xu et al. 2007). *Rhodococcus* sp. OC_3C OTU2 was consistently found in the OC-degrading enrichment cultures and may have a role in the lower degradation pathway of OC but is not able to perform the initial hydrolysis of OC (Figure S8). Interestingly, five different *Microbacterium* strains were isolated, all of which were able to degrade different UV filters (Fig. 1). These five strains were decidedly different from each other, with sequence identity from 93.7 to 98.20%, and closely related to cultured strains isolated from the phyllosphere of grasses (Behrendt et al. 2001) for strain BM_17A and homemade compost (Vaz-Moreira et al. 2009) for strain HS_40C, as well as medical samples (Schumann et al. 1999). These strains were not found by Illumina sequencing as OTUs; however, this may be due to sequencing bias. Indeed, upon further investigation of the primers previously used for Illumina sequencing (Fagervold and Lebaron 2022). It was discovered that there is a mismatch for many of the *Microbacteria* (but not *Gordonia* OC-13I-OTU1 and *Rhodococcus* OC_3C_OTU2). This mismatch may cause a bias in the sequencing results and result in an underestimation of the amount of *Microbacteria* in the enrichment cultures*.*

Most of the isolated strains belonged to the phylum *Pseudomonadota.* Within this phylum, many *Alphaproteobacteria*, mostly belonging to the family *Sphingomonadaceae,* exhibited degradation activity toward BM, OC and HS (Fig. 1). Furthermore, a previously described S*phingomonas* strain was shown to readily degrade BP3 (Fagervold et al. 2021). The OC-degrading *Sphingopyxis strain* OC_4D has been described previously (Fagervold and Lebaron 2022). Generally, this group of bacteria is known for their biodegradation capabilities of aromatic compounds and, thus, has great potential for the biodegradation of organic UV filters. For example, BM_18B is only 3 bp different (over 1370-bp 16S rRNA gene) from a *Sphingobium phenoxybenzoativorans* strain SC_3, which degrades 2-phenoxybenzoic acid (Cai et al. 2015). The other strains were less similar to the characterized strains (Table 3).

Although *Pseudomonas* species were present in the enrichments degrading BM, HS, and ES in significant amounts, only two of the four strains tested exhibited degradation capacity. Indeed, even though OTU 33 was present in ES-degrading cultures at a relative abundance of almost 50% (Fagervold and Lebaron 2022), the corresponding strain ES_58E (OTU33) did not degrade ES. Instead, strain OC_8G (same as ES_54_D), representing OTU 32, degraded ES. Generally, *Pseudomonas* strains are found in various environments and are sometimes very close to human pathogens; for example, strain BM_21C was only 3 bp different from *Pseudomonas aeruginosa* strain DSM 50071 and the type strain of *P. aeruginosa*.

Within the *Betaproteobacteria,* several strains belonged to the genus *Hydrogenophaga*. Bacteria from this genus were detected in all the cultures degrading OC, BM, HS, and ES to some extent (Fagervold and Lebaron 2022). However, their exact role is unclear. The strains OC_2B (OTU24) and OC_1A (OTU27) did not degrade OC, but strain OC_1A (OTU27) did degrade BM and may indeed have a role in BM degradation, as this OTU was present in significant amounts in BM-degrading cultures. Strain OC_1A (OTU27) was 98.98% identical to *Hydrogenophaga intermedia* strain S1 (NR_024856), which has been shown to be able to degrade 4-aminobenzenesulfonate (Contzen et al. 2000) and has been shown to have dioxygenase genes (Contzen et al. 2001) perhaps needed for the degradation of BM and possibly for the lower degradation pathway of OC (ST1 and Figure S8). Indeed, the *Hydrogenophaga* strains are “only” approximately 97–98.5% identical to each other, so they are clearly different ecotypes/species. Thus, different ecotypes/species of the genus *Hydrogenophaga* were enriched in different cultures. Interestingly, the 16S rRNA gene sequence of strain OC_2B (OTU24) is only 4 bp different (1455 bp) from that of the *Hydrogenophaga electricum* strain AR20, which has been shown to be able to oxidize hydrogen in a pure culture microbial fuel cell (Kimura and Okabe 2013). Therefore, strain OC_2B may have a role in the intermediary metabolism of OC degradation. However, it should be noted that not all species in the *Hydrogenophaga* genus can utilize hydrogen (Gan et al. 2017).

Additionally, within the *Betaproteobacteria*, several strains were able to degrade OC, HS, and ES, namely, *Pigmentiphaga* OC_11K, *Acidovorax* ES_53, and *Comamonas* ES_S3. None of these strains was very similar to their closest cultured relatives. *Pigmentiphaga* OC_11K was 98.8% similar to a strain involved in the degradation of azo dyes (Blümel et al. 2001), *Acidovorax* ES_53 was approximately 99.5% identical to strains from different environments, including clinical samples (Vaneechoutte et al. 2013), and *Comamonas* ES _S3 was only 97.2% similar to the closest culture strain. Finally, the *Chitinophaga* strain OC_16J was only 97.3% identical to the nearest *Chitinophaga* strain isolated from soil. Generally, this group is known for its organic matter decomposition capability, but the exact role this strain has in these enrichment cultures is unknown.

### Microbial consortia dynamics

The in-house synthetic defined consortium was developed through a “bottom-up” approach (Liang et al. 2022), where different isolates were assembled to obtain a specific function, in this case, the degradation of organic UV filters. Indeed, this synthetic consortium successfully degraded four organic UV filters to a great degree. However, it should be noted that the presence of a strain 100% identical to *Sphingomonas wittichii* strain BP14P, namely strain HS_37A, in the in-house consortium did not result in the degradation of BP3. This is despite the fact that *Sphingomonas wittichii* strain BP14P has shown to degrade BP3 previously (Fagervold et al. 2021). The reason for this discrepancy was originally hypothesized to be because this strain was not present in the appropriate amounts and/or that this strain was outcompeted by other microorganisms present. Generally, for the maintenance of a consortium, there will be competition for resources between the different taxa, and one species tends to dominate over time (Liang et al. 2022). However, it may be that HS_37A is not capable of degrading BP3 and has different degradation capabilities than *Sphingomonas wittichii* strain BP14P even though the 16S is 100% identical.

The stable enrichment cultures, from which the isolates were derived, can be seen as being assembled using a “top-down” strategy. These cultures have been transferred over 20 times with a single organic UV filter as the sole carbon source and have given rise to specific enrichment cultures that are relatively efficient in degrading specific organic UV filters. However, the difference is that these “top-down” enrichments were specific for one UV filter, but the defined synthetic consortia could degrade four different UV filters. However, the different enrichment cultures have not been tested with other organic UV filters; thus, the redundancy in these more complex consortia is unknown. Indeed, it is interesting that the transfers selected for several different strains capable of degradation; thus, there was certainly some functional redundancy in these cultures and it seems like there were some selective pressure to keep these specific microorganisms present. However, with time, one may lose the “auxiliary bacteria,” i.e., bacteria that may not be directly involved in the degradation process but can indirectly impact the process (Li et al. 2022). Indeed, one positive point for the “bottom-up” approach is that it is easier to maintain over the long term, as one can assemble the consortium when the need arises. Thus, the problem of stability one can have with “top-down” bacterial consortia is circumvented.

The commercially available consortia, although not at all “optimized” for organic UV filter degradation, still degrade some of the UV filters. Mix 4, which contained *Bacillus* strains and some fungal strains (Table 1), did show some degradation of ES and HS. Furthermore, ES showed some degradation by mix 1 and mix 5. Hence, it seems that ES may be an easier target for microorganisms than many of the other organic UV filters tested here. It should also be noted that contrary to experiments carried out with the in-house synthetic consortium, the commercial consortium’s ability to degrade UV filters was evaluated for several UV filters at once; a possible cocktail effect of UV filters may be observed.

### Possible hurdles for biodegradation

It is clear that the presence of different organic UV filters enriched for different degrading microbial communities. Furthermore, different strains were isolated from different enrichment cultures. It is thus evident that not all microorganisms are capable of utilizing organic UV filters as carbon sources or can transform them co-metabolically. It then follows that the degradation of specific organic UV filters requires specific enzymes from specific microorganisms. However, the exact microorganisms/enzymes needed are currently still unclear. We show here that four to six different microorganisms are capable of degrading OC, BM, HS, and ES (Table 2; Fig. 1). However, it is difficult to assign specific roles to the different strains, mainly because one has to perform more in-depth experiments with the strains to obtain better knowledge of the enzymes involved and the exact degradation pathways. One good example is the work performed by Baek and colleagues for the further elucidation of the BP3 degradation pathway by strain *Rhodococcus oxybenzonivorans* sp. S2-17 (Baek et al. 2022b).

The microorganisms/enzymes appear to be specific according to the different organic UV filters, but as shown by Table 4, the first steps in the predicted degradation pathways seem to require similar enzymatic activities in many cases. This suggests that it might not be the presence/absence of specific enzymes that makes the recalcitrant organic UV filter non-biodegradable. In support of this is the fact that stimulation attempts by the addition of easily degradable UV filters, meaning that the enzymatic activities are induced and had no effect on the degradation of the recalcitrant UV filters. In these experiment, the organic UV filters were effectively added to the cultures as solids (after solubilization in, followed by evaporation of, acetone), due to the low solubility of many of the filters (Table 4). Indeed, for OC, for example, one can clearly observe droplets (OC has a molasses like consistency) at day 0, but these droplets disappear during incubation and, as shown in Fig. 2, OC is clearly available for biodegradation, with 90% degradation after 12 days. The same can be said for HS, which has a relatively low solubility, but is clearly degraded. This being said, the recalcitrant organic UV filters have extreme low solubility, so this is most probable the reason why they are not degraded. One other factor is the size; all recalcitrant UV filters have a molecular weight > 600 Da, except DHHB (Table 4).

Other hurdles for biodegradation could be possible toxicity of the UV filters on the microbial community. While none of the UV filters has been deemed toxic to sludge microorganisms, other tests, like the Luminescent Bacteria tests with *Vibrio fischeri* (Strotmann et al. 2020), would be a useful test in this regard. BP3 has been shown to inhibit the growth of *Vibrio fischeri* (Zhang et al. 2017), but otherwise, few studies have been reported. Interestingly, Lozano et al. (2020b) used 27 different marine bacterial strains to test different UV filters for growth inhibition. They showed that BP3, OC, and HS inhibited growth of only a couple of marine bacterial strains (Lozano et al. 2020b). Thus, UV filters does not seem to be acutely toxic to microorganisms, at least not at concentrations found in the environment.

### Comparison to OECD biodegradation tests

Here, we have assessed the biodegradability of UV filters by following their decrease in microcosms with time using specific consortia. These experiments can be compared to the commonly used OECD test for assessing the biodegradability of chemicals (see Strotmann et al. (2023) for a recent review) in some ways but with caveats. For example, the commonly used OECD readily biodegradability tests (RBT) often follows the degradation of a chemical indirectly by for measuring the production of CO_2_, the depletion of O_2_ or the removal of dissolved organic carbon (DOC) from the system. Thus, for example if one would test mixtures of different compounds, one would not know what constituents are degraded. Furthermore, the concentrations of the test substance used in the RBT tests are generally comparable to what has been used here, around 100 mg/L. The main difference, however, is that the inoculum used here, specific microbial consortia, would not be acceptable under the OECD guidelines. The RBT test does not allow for pre-adapted inoculum to be used. This being said, looking at the degradation curves of OC, ES and HS (Fig. 2), it is clear that they are degraded over 70% over a 10-day window, which is the pass level for RBT tests using DOC removal. BM was degraded around 50% from day 0 to day 12. Un-enriched sludge has previously been shown to degrade BP3 completely after 20 days (Fagervold et al. 2021); the same was seen for BM and ES, while OC and HS degraded less than 50% after 20 days in un-enriched sludge (Fagervold and Lebaron 2022).

Official OECD test has been performed on some of the UV filters targeted in this work. BM and OC have been deemed not biodegradable and poorly biodegradable, respectively, according to several OECD tests (ECHA 2023a, 2023b) and reviewed by Duis et al. (2022)). HS and ES on the other hand have been deemed readily biodegradable (ECHA 2023c, 2023d). In addition, an official RBT has been recently reported on BP3 using both WWTP sludge and river water as inocula showing that BP3 is indeed readily biodegradable (Carstensen et al. 2023).

## Conclusions

Several different consortia of microorganisms have been tested, demonstrating that the organic UV filters BP3, OC, HS, ES, and BM are degraded by a seemingly specific set of microorganisms. This degradation capability is not a universal feature in all microorganisms. Consequently, degradation of organic UV filters in the environment may be “site specific.” However, some of the filters could be deemed “biodegradable” if one uses an inoculum from WWTPs. This could be the case for OC and BM, which is poorly degradable according the ECHA, but has shown degradation in both non-enriched sludge and by specific a specific consortium. Organic UV filters comprise compounds with diverse structures and different chemical characteristics; thus, there is not necessarily a commonality of microorganisms/enzymes that can degrade all organic UV filters. Nevertheless, the expansion of the quantity of bacterial isolates that are capable of degrading these compounds shows promise for the future.

It is clear that several “new-generation” organic UV filters are not degraded by WWTP sludge (Fagervold and Lebaron 2022) or the in-house consortia described here. Furthermore, there were no signs of degradation by the consortia from Greencell, several of which also contained fungi. The reason for this lack of degradation is probably size and very low solubility, making these compounds poorly bioavailable to microorganisms. Conversely, these same characteristics may render them not bioavailable to cause toxic effects, as very little data are available on the toxic effects of these recalcitrant filters.

### Supplementary Information

Below is the link to the electronic supplementary material.

Supplementary file1 (PDF 649 KB)

## Data Availability

Sequences have been submitted to Genbank under accession numbers OP985055-OP985077 and the strains are available at the Banyuls Bacterial Culture Collection (https://banyuls-bacterial-culture-collection.fr/).

## References

[CR1] Alonso MB, Feo ML, Corcellas C (2015). Toxic heritage: maternal transfer of pyrethroid insecticides and sunscreen agents in dolphins from Brazil. Environ Pollut.

[CR2] Badia-Fabregat M, Rodríguez-Rodríguez CE, Gago-Ferrero P (2012). Degradation of UV filters in sewage sludge and 4-MBC in liquid medium by the ligninolytic fungus Trametes versicolor. J Environ Manage.

[CR3] Baek JH, Baek W, Jeong SE et al (2022a) Rhodococcus oxybenzonivorans sp. nov., a benzophenone-3-degrading bacterium, isolated from stream sediment. Int J Syst Evol Microbiol 72(6). 10.1099/ijsem.0.005433 10.1099/ijsem.0.005433 35704462

[CR4] Baek JH, Kim KH, Lee Y et al (2022b) Elucidating the biodegradation pathway and catabolic genes of benzophenone-3 in Rhodococcus sp. S2–17. Environ Pollut 299:118890. 10.1016/j.envpol.2022.118890 10.1016/j.envpol.2022.118890 35085657

[CR5] Behrendt U, Ulrich A, Schumann P (2001). Description of Microbacterium foliorum sp. nov. and Microbacterium phyllosphaerae sp. nov., isolated from the phyllosphere of grasses and the surface litter after mulching the sward, and reclassification of Aureobacterium resistens (Funke et al. 1998) as Microbacterium resistens comb. nov. Int J Syst Evol Microbiol.

[CR6] Bhatt P, Gangola S, Bhandari G (2021). New insights into the degradation of synthetic pollutants in contaminated environments. Chemosphere.

[CR7] Blümel S, Mark B, Busse HJ (2001). Pigmentiphaga kullae gen. nov., sp. nov., a novel member of the family Alcaligenaceae with the ability to decolorize azo dyes aerobically. Int J Syst Evol Microbiol.

[CR8] Cai S, Shi C, Zhao J-D (2015). Sphingobium phenoxybenzoativorans sp. nov., a 2-phenoxybenzoic-acid-degrading bacterium. Int J Syst Evol Microbiol.

[CR9] Carstensen L, Beil S, Schwab E (2023). Primary and ultimate degradation of benzophenone-type UV filters under different environmental conditions and the underlying structure-biodegradability relationships. J Hazard Mater.

[CR10] Chisvert A, Salvador A, Salvador A, Chisvert A (2007). 3.1 - UV filters in sunscreens and other cosmetics. regulatory aspects and analytical methods. Analysis of cosmetic products.

[CR11] Contzen M, Moore ERB, Blümel S (2000). Hydrogenophaga intermedia sp. nov., a 4-aminobenzene-sulfonate Degrading Organism. Syst Appl Microbiol.

[CR12] Contzen M, Bürger S, Stolz A (2001). Cloning of the genes for a 4-sulphocatechol-oxidizing protocatechuate 3,4-dioxygenase from Hydrogenophaga intermedia S1 and identification of the amino acid residues responsible for the ability to convert 4-sulphocatechol. Mol Microbiol.

[CR13] Dereeper A, Guignon V, Blanc G (2008). Phylogeny.fr: robust phylogenetic analysis for the non-specialist. Nucleic Acids Res.

[CR14] Díaz-Cruz MS, Molins-Delgado D, Serra-Roig MP (2019). Personal care products reconnaissance in EVROTAS river (Greece): water-sediment partition and bioaccumulation in fish. Sci Total Environ.

[CR15] Downs CA, Kramarsky-Winter E, JohnE F (2014). Toxicological effects of the sunscreen UV filter, benzophenone-2, on planulae and in vitro cells of the coral, Stylophora pistillata. Ecotoxicology.

[CR16] Duis K, Junker T, Coors A (2022). Review of the environmental fate and effects of two UV filter substances used in cosmetic products. Sci Total Environ.

[CR17] ECHA, 2023a Information on registered substances. Octocrilene. Available at: European Chemicals Agency (2023) (accessed 12 October 2023) https://echa.europa.eu/de/registration-dossier/-/registered-dossier/14858/5/3/2

[CR18] ECHA, 2023b Information on Registered Substances. 1-[4-(1,1-dimethylethyl)phenyl]-3-(4-methoxyphenyl)propane-1,3-dione. Available at: European Chemicals Agency (2023) (accessed 12 October 2023) https://echa.europa.eu/de/registration-dossier/-/registered-dossier/14835/5/3/3

[CR19] ECHA, 2023c Information on registered substances. Homosalate. Available at: European Chemicals Agency (2023) (accessed 12 October 2023) hhttps://echa.europa.eu/registration-dossier/-/registered-dossier/13246/5/3/2

[CR20] ECHA, 2023d Information on registered substances. 2-ethylhexyl salicylate. Available at: European Chemicals Agency (2023) (accessed 12 October 2023) https://echa.europa.eu/registration-dossier/-/registered-dossier/14203/5/3/2

[CR21] Egambaram OP, Kesavan Pillai S, Ray SS (2020). Materials science challenges in skin UV protection: a review. Photochem Photobiol.

[CR22] EU (2021) Cosmetic ingredient database (Cosing). List of UV filters allowed in cosmetic products. Annex VI. http://ec.europa.eu/growth/tools-databases/cosing/pdf/COSING_Annex %20VI_v2.pdf

[CR23] Fagervold SK, Lebaron P (2022). Evaluation of the degradation capacity of WWTP sludge enrichment cultures towards several organic UV filters and the isolation of octocrylene-degrading microorganisms. Sci Total Environ.

[CR24] Fagervold SK, Rodrigues AS, Rohée C (2019). Occurrence and environmental distribution of 5 UV filters during the summer season in different water bodies. Water Air Soil Pollut.

[CR25] Fagervold SK, Rohée C, Rodrigues AMS (2021). Efficient degradation of the organic UV filter benzophenone-3 by Sphingomonas wittichii strain BP14P isolated from WWTP sludge. Sci Total Environ.

[CR26] Fel J-P, Lacherez C, Bensetra A (2019). Photochemical response of the scleractinian coral Stylophora pistillata to some sunscreen ingredients. Coral Reefs.

[CR27] Fisher MM, Triplett EW (1999). Automated approach for ribosomal intergenic spacer analysis of microbial diversity and its application to freshwater bacterial communities. Appl Env Microbiol.

[CR28] Fujii K, Kikuchi S (2005). Degradation of benzophenone, a potential xenoestrogen, by a yeast isolated from the activated sludge of a sewage treatment plant in Hokkaido. World J Microbiol Biotechnol.

[CR29] Gago-Ferrero P, Díaz-Cruz MS, Barceló D (2015). UV filters bioaccumulation in fish from Iberian river basins. Sci Total Environ.

[CR30] Gan HM, Lee YP, Austin CM (2017). Nanopore long-read guided complete genome assembly of Hydrogenophaga intermedia, and genomic insights into 4-aminobenzenesulfonate, p-aminobenzoic acid and hydrogen metabolism in the genus Hydrogenophaga. Front Microbiol.

[CR31] Gao J, Ellis LB, Wackett LP (2010). The University of Minnesota Biocatalysis/Biodegradation Database: improving public access. Nucleic Acids Res.

[CR32] Gilbert E, Pirot F, Bertholle V (2013). Commonly used UV filter toxicity on biological functions: review of last decade studies. Int J Cosmet Sci.

[CR33] Hany J, Nagel R (1995). Detection of sunscreen agents in human breast-milk. Dtsch Lebensm-Rundsch.

[CR34] He T, Tsui MMP, Tan CJ (2019). Comparative toxicities of four benzophenone ultraviolet filters to two life stages of two coral species. Sci Total Environ.

[CR35] Jin C, Geng Z, Pang X (2019). Isolation and characterization of a novel benzophenone-3-degrading bacterium Methylophilus sp. strain FP-6. Ecotoxicol Environ Saf.

[CR36] Kang D, Jacquiod S, Herschend J et al (2020) Construction of simplified microbial consortia to degrade recalcitrant materials based on enrichment and dilution-to-extinction cultures. Front Microbiol 10:3010. 10.3389/fmicb.2019.03010 10.3389/fmicb.2019.03010 PMC6968696 31998278

[CR37] Kimura Z, Okabe S (2013). Hydrogenophaga electricum sp. nov., isolated from anodic biofilms of an acetate-fed microbial fuel cell. J Gen Appl Microbiol.

[CR38] Letunic I, Bork P (2021). Interactive Tree Of Life (iTOL) v5: an online tool for phylogenetic tree display and annotation. Nucleic Acids Res.

[CR39] Li X, Lu C, Dai Y et al (2022) Characterizing the microbial consortium L1 capable of efficiently degrading chlorimuron-ethyl via metagenome combining 16S rDNA sequencing. Front Microbiol 13:912312. 10.3389/fmicb.2022.912312 10.3389/fmicb.2022.912312 PMC9260513 35814706

[CR40] Liang Y, Ma A, Zhuang G (2022) Construction of Environmental Synthetic Microbial Consortia: based on engineering and ecological principles. Front Microbiol 13:829717. 10.3389/fmicb.2022.829717 10.3389/fmicb.2022.829717 PMC8905317 35283862

[CR41] Liu Y-S, Ying G-G, Shareef A, Kookana RS (2013). Degradation of six selected ultraviolet filters in aquifer materials under various redox conditions. Groundw Monit Remediat.

[CR42] Lozano C, Givens J, Stien D (2020). Bioaccumulation and toxicological effects of UV-filters on marine species. The handbook of environmental chemistry.

[CR43] Lozano C, Matallana-Surget S, Givens J (2020). Toxicity of UV filters on marine bacteria: combined effects with damaging solar radiation. Sci Total Environ.

[CR44] Lu S, Long F, Lu P (2018). Benzophenone-UV filters in personal care products and urine of schoolchildren from Shenzhen, China: exposure assessment and possible source. Sci Total Environ.

[CR45] Miller IB, Pawlowski S, Kellermann MY (2021). Toxic effects of UV filters from sunscreens on coral reefs revisited: regulatory aspects for “reef safe” products. Environ Sci Eur.

[CR46] Molins-Delgado D, Máñez M, Andreu A (2017). A potential new threat to wild life: presence of UV filters in bird eggs from a preserved area. Environ Sci Technol.

[CR47] Osterwalder U, Sohn M, Herzog B (2014). Global state of sunscreens. Photodermatol Photoimmunol Photomed.

[CR48] Ramos S, Homem V, Alves A, Santos L (2016). A review of organic UV-filters in wastewater treatment plants. Environ Int.

[CR49] Schlumpf M, Kypke K, Wittassek M (2010). Exposure patterns of UV filters, fragrances, parabens, phthalates, organochlor pesticides, PBDEs, and PCBs in human milk: Correlation of UV filters with use of cosmetics. Chemosphere.

[CR50] Schumann P, Rainey FA, Burghardt J (1999). Reclassification of Brevibacterium oxydans (Chatelain and Second 1966) as Microbacterium oxydans comb. nov. Int J Syst Evol Microbiol.

[CR51] Strotmann U, Pastor Flores D, Konrad O, Gendig C (2020). Bacterial toxicity testing: modification and evaluation of the luminescent bacteria test and the respiration inhibition test. Processes.

[CR52] Strotmann U, Thouand G, Pagga U (2023). Toward the future of OECD/ISO biodegradability testing-new approaches and developments. Appl Microbiol Biotechnol.

[CR53] Suleiman M, Schröder C, Kuhn M (2019). Microbial biofilm formation and degradation of octocrylene, a UV absorber found in sunscreen. Commun Biol.

[CR54] Vaneechoutte M, Janssens M, Avesani V (2013). Description of Acidovorax wautersii sp. nov. to accommodate clinical isolates and an environmental isolate, most closely related to Acidovorax avenae. Int J Syst Evol Microbiol.

[CR55] Varrella S, Danovaro R, Corinaldesi C (2022). Assessing the eco-compatibility of new generation sunscreen products through a combined microscopic-molecular approach. Environ Pollut.

[CR56] Vaz-Moreira I, Lopes AR, Faria C (2009). Microbacterium invictum sp. nov., isolated from homemade compost. Int J Syst Evol Microbiol.

[CR57] Xu J-L, He J, Wang Z-C (2007). Rhodococcus qingshengii sp. nov., a carbendazim-degrading bacterium. Int J Syst Evol Microbiol.

[CR58] Zhang Q, Ma X, Dzakpasu M, Wang XC (2017). Evaluation of ecotoxicological effects of benzophenone UV filters: luminescent bacteria toxicity, genotoxicity and hormonal activity. Ecotoxicol Environ Saf.

[CR59] Zhang T, Zhang H (2022) Microbial consortia are needed to degrade soil pollutants. Microorganisms 10:261. 10.3390/microorganisms10020261 10.3390/microorganisms10020261 PMC8874626 35208716

