# Supplementary material for: Microbial consortia degrade several widely used organic UV filters, but a number of hydrophobic filters remain recalcitrant to biodegradation
Source: Environ Sci Pollut Res Int. 2023 Nov 27;30(60):125931–46. doi: 10.1007/s11356-023-31063-w (PMC10754744; doi:10.1007/s11356-023-31063-w)
Supplement: Supplementary file 1 — Supplementary file1 (PDF 649 KB) [file 11356_2023_31063_MOESM1_ESM.pdf]

Supplementary material

Supplemental Text 1.

Possible degradation pathways of BP3, BM, ES, HS and OC

BP3 degradation pathway

The degradation pathway for BP3 has been previously proposed, based upon the pathway prediction system and the presence of certain intermediates (Jin et al. 2019; Fagervold et al. 2021). Further, the BP3 degradation pathway in *Rhodococcus* strain S2-17 has been investigated more in detail with transcriptomic analyses and biochemical and genetic studies (Baek et al. 2022). The degradation starts by a demethylation reaction to BP1, catalyzed by Cytochrome P450, ferredoxin, FAD dependent oxidoreductase. BP1 is further degraded by hydroxylation and then by dioxygenation. The end products are fed into the benzoate degradation pathway and then into the TCA cycle (general metabolism). The pathway prediction system did indeed predict many of the reactions and intermediates further verified by Baek and colleagues (Baek et al. 2022).

First predicted steps in the BM degradation pathway

Similar to BP3, the predicted first degradation step for BM is either a demethylation by monooxygenase [bt0023], figure S5 or a hydroxylation of a secondary aliphatic carbon by a monooxygeanse [bt0242]. These intermediates are then predicted to be transformed by the action of various enzymes, including monooxygenase and dehydrogenases to compounds that will feed into the central carbohydrate metabolism. However, the exact pathway has not been determined. The first steps are similar to that of the BP3 predicted degradation pathway.

First predicted steps in the ES degradation pathway

The predicted degradation of ES starts by esterase or hydrolase activity, resulting in 2-hydroxy-benzoate (Salicylate) and 2-ethylhexan-1-ol (Figure S6). Salicylate can then be transformed into either catechol or gentisate by the action of hydroxylases, and are then converted into common intermediates in the general metabolism of microorganisms (TCA cycle and glycolysis). 2-Ethylhexan-1-ol can be converted to 2-ethylhexanal by a dehydrogenase and then to 2-ethylhexanoate, also by a dehydrogenase. 2-ethylheaxanoate can then be further oxidized in several possible positions (not shown) by various monooxygenases.

First predicted steps in the HS degradation pathway

Similar to the ES predicted pathway, the first predicted reaction in HS degradation pathway involves a esterase / hydrolase activity, which results in salicylate and 3,3,5-trimethyl-1-cyclohexanol by (Figure S7). The salicylate degradation pathway is described for ES. 3,3,5-Trimethyl-1-cyclohexanol is predicted to be transformed into 3,3,5-Trimethyl-1-cyclohexanone, and then oxidized to 4,6,6-Trimethyloxepan-2-one by a monooxygenase. The further pathway most probably involves further oxidation reactions but the exact reactions and products are unclear.

First predicted steps in the OC degradation pathway

The exact pathways are highly uncertain for OC as there are a lot of different options according the pathway prediction system. Furthermore, many OC degradation products were detected in chlorination treatment experiments (Medici et al. 2022) and it has also been shown that OC can be converted to Benzophenone through an "aging" process (Downs et al. 2021) involving a "Retro-aldol condensation reaction" (Figure S8). However this process is most probably not microbially mediated, but can perhaps still be happening in the enrichment cultures. According to the prediction system, the most probable microbial mediated process starts with an esterase/hydrolase action resulting in the 2-Ethylhexan-1-ol and a compound (unknown name) with the nitrile group. 2-Ethylhexan-1-ol was also a product of the first reaction for ES, see over for further description. From here the possible pathways are many, but at some point the nitrile group will be converted to a carboxylate group by a nitrilase. There are many predicted intermediates, including Benzophenone. Through the action of various oxygenases and hydrolases the end predicted products are compounds like oxalate and pyruvate, which are intermediates in the general bacterial metabolism

References:

Baek JH, Kim KH, Lee Y, et al (2022) Elucidating the biodegradation pathway and catabolic genes of benzophenone-3 in Rhodococcus sp. S2-17. Environ Pollut 299:118890. https://doi.org/10.1016/j.envpol.2022.118890

Downs CA, DiNardo JC, Stien D, et al (2021) Benzophenone Accumulates over Time from the Degradation of Octocrylene in Commercial Sunscreen Products. Chem Res Toxicol 34:1046–1054. https://doi.org/10.1021/acs.chemrestox.0c00461

Fagervold SK, Rohée C, Rodrigues AMS, et al (2021) Efficient degradation of the organic UV filter benzophenone-3 by Sphingomonas wittichii strain BP14P isolated from WWTP sludge. Sci Total Environ 758:143674. https://doi.org/10.1016/j.scitotenv.2020.143674

Jin C, Geng Z, Pang X, et al (2019) Isolation and characterization of a novel benzophenone-3-degrading bacterium Methylophilus sp. strain FP-6. Ecotoxicol Environ Saf 186:109780. https://doi.org/10.1016/j.ecoenv.2019.109780

Medici A, Saviano L, Siciliano A, et al (2022) Octocrylene: From Sunscreens to the Degradation Pathway during Chlorination Processes: Formation of Byproducts and Their Ecotoxicity Assessment. Molecules 27:5286

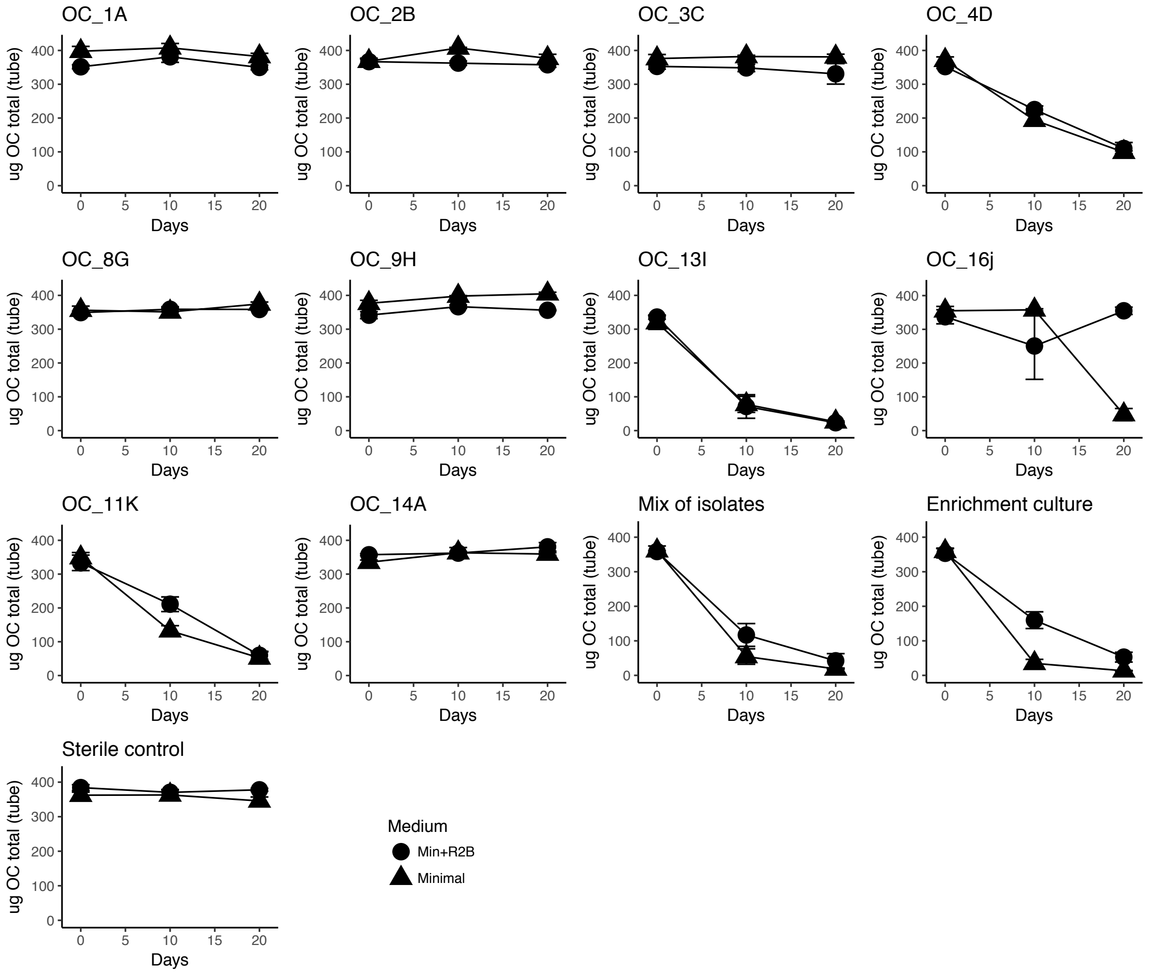

Figure S1. Degradation assays of OC performed on different isolates and appropriate controls. The Y-axis depicts total amount of OC in tubes. Triangles represent OC in cultures grown in minimal media and circles represent OC in cultures grown with minimal media with added 20% R2B.

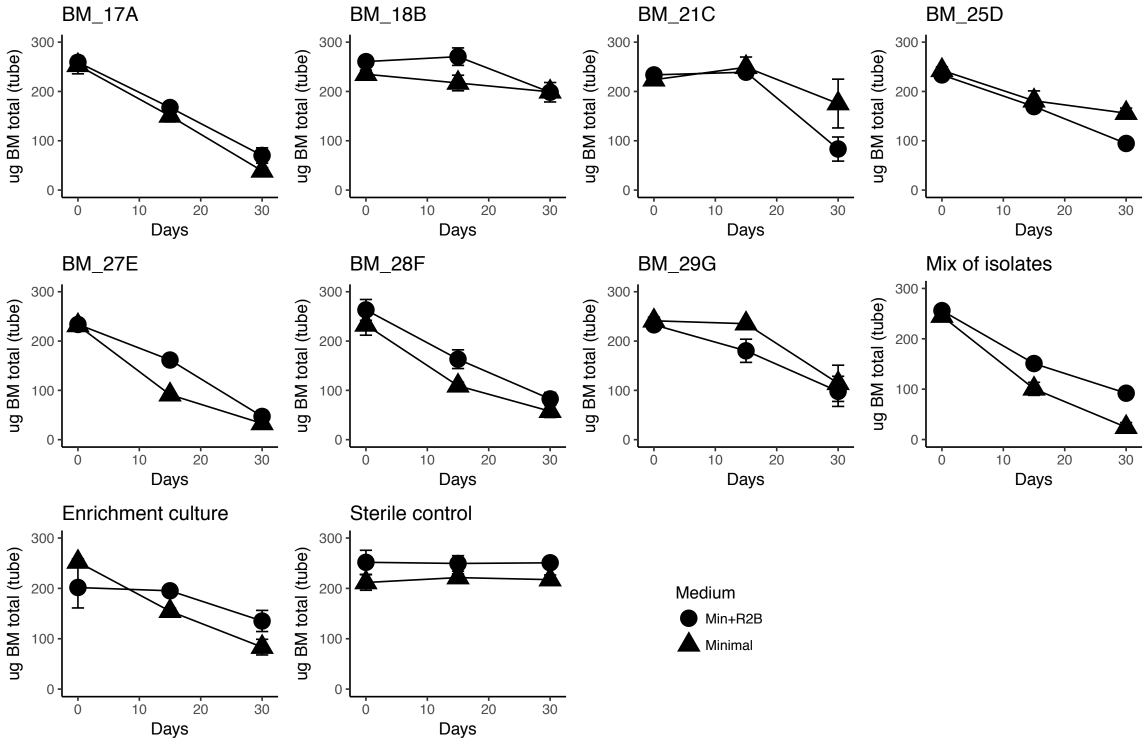

Figure S2. Degradation assays of BM performed on different isolates and appropriate controls. The Y-axis depicts total amount of BM in tubes. Triangles represent BM in cultures grown in minimal media and circles represent OC in cultures grown with minimal media with added 20% R2B.

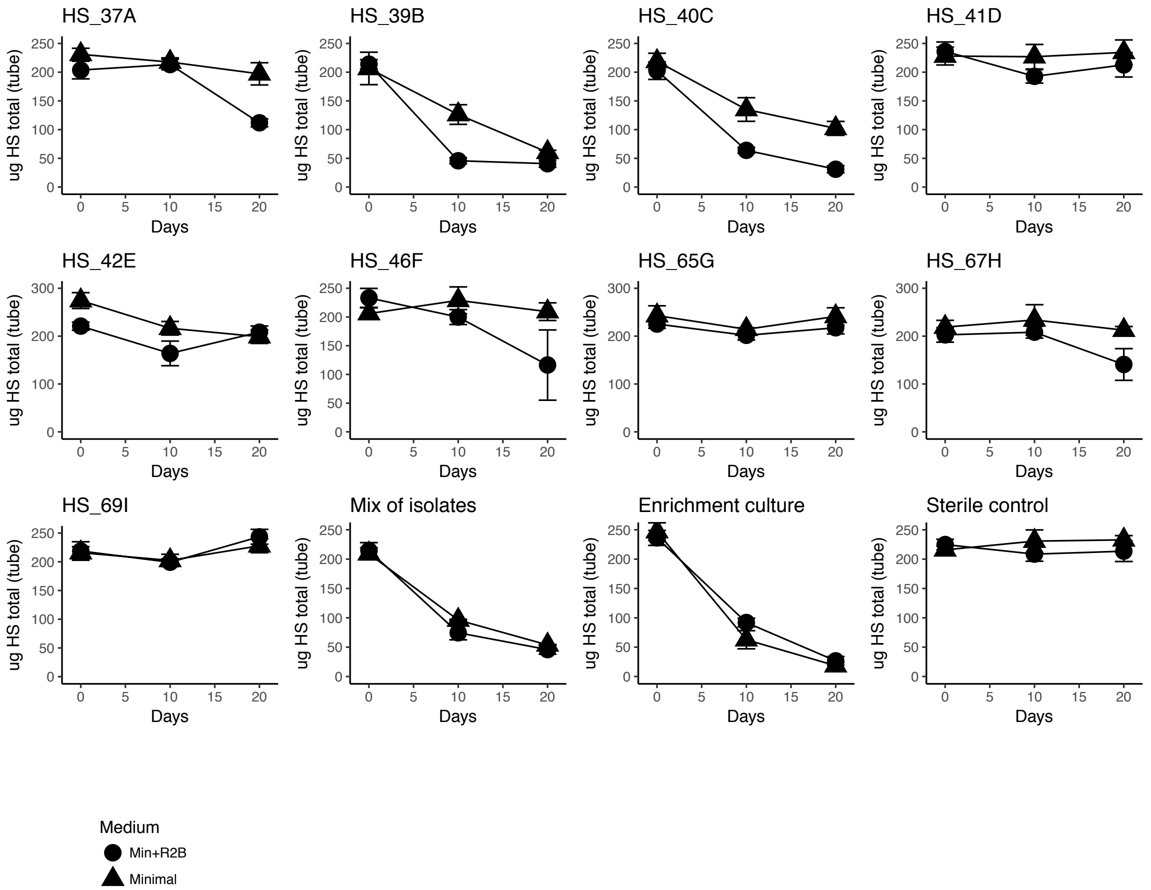

Figure S3. Degradation assays of HS performed on different isolates and appropriate controls. The Y-axis depicts total amount of HS in tubes. Triangles represent HS in cultures grown in minimal media and circles represent HS in cultures grown with minimal media with added 20% R2B.

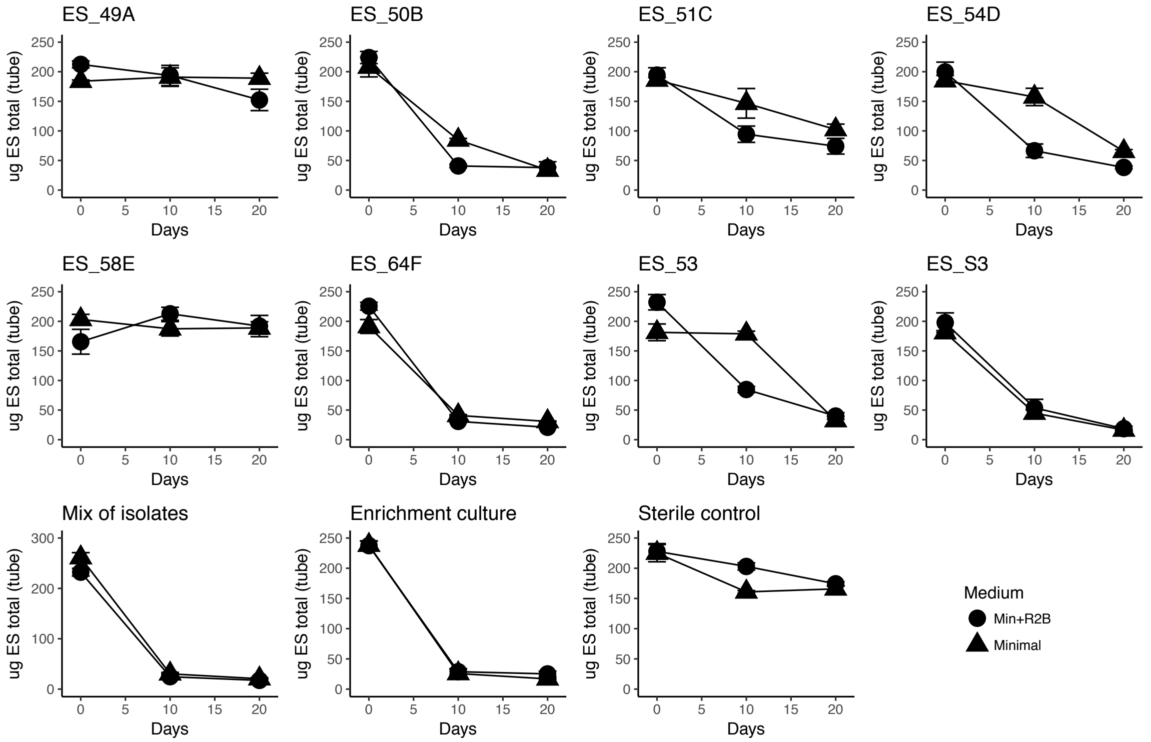

Figure S4. Degradation assays of ES performed on different isolates and appropriate controls. The Y-axis depicts total amount of HS in tubes. Triangles represent ES in cultures grown in minimal media and circles represent HS in cultures grown with minimal media with added 20% R2B.

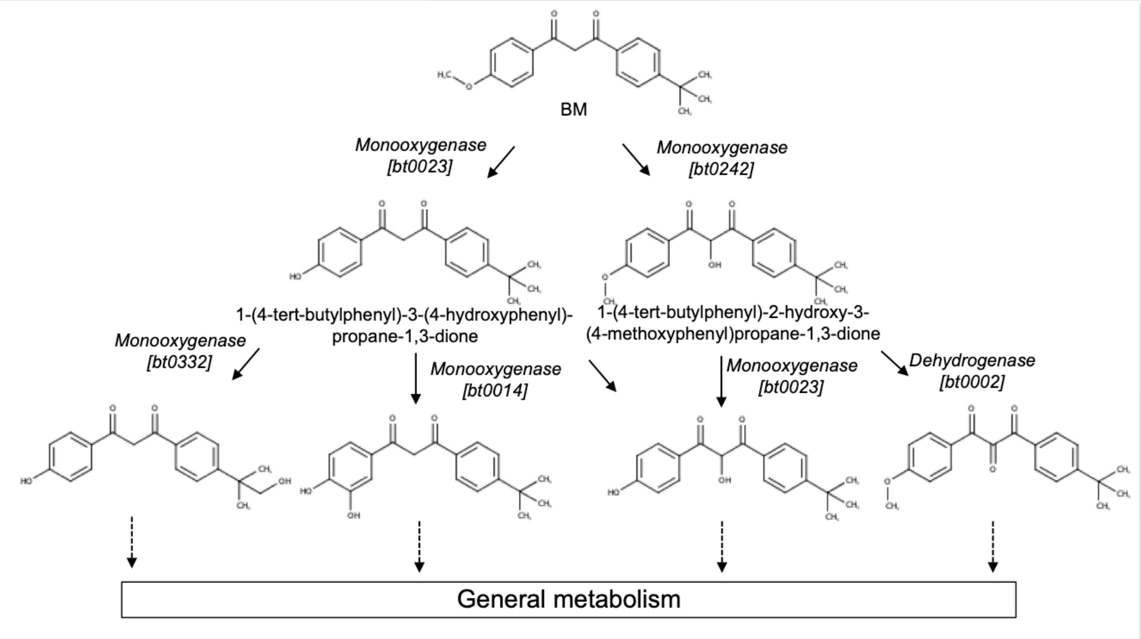

Figure S5. Predicted first steps in a proposed BM degradation pathway. The text between [] refers to the “reaction rule” of the prediction system (http://eawag-bbd.ethz.ch/predict/)

.

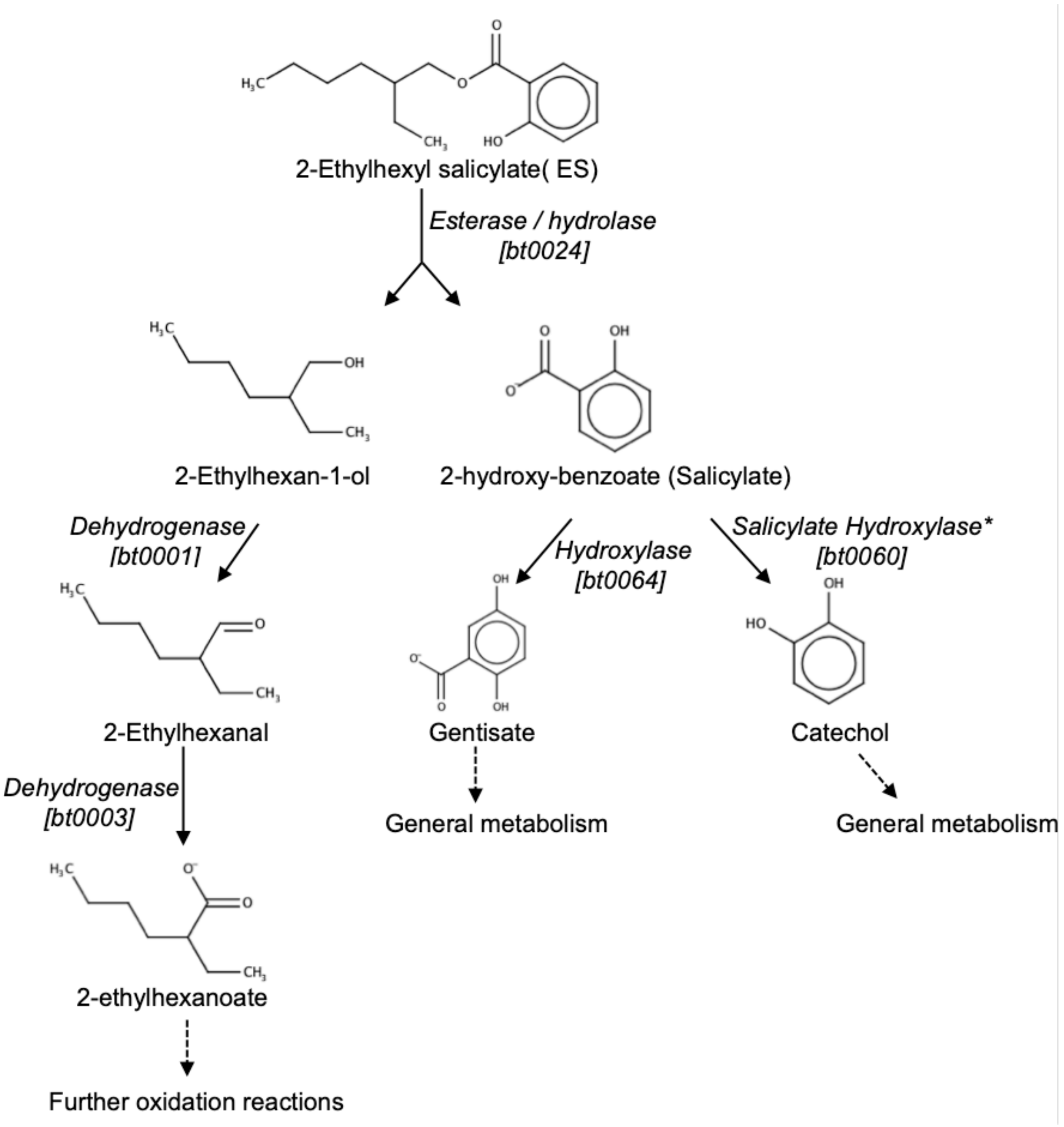

Figure S6. Predicted first steps in a proposed ES degradation pathway. The text between [] refers to the “reaction rule” of the prediction system (http://eawag-bbd.ethz.ch/predict/)

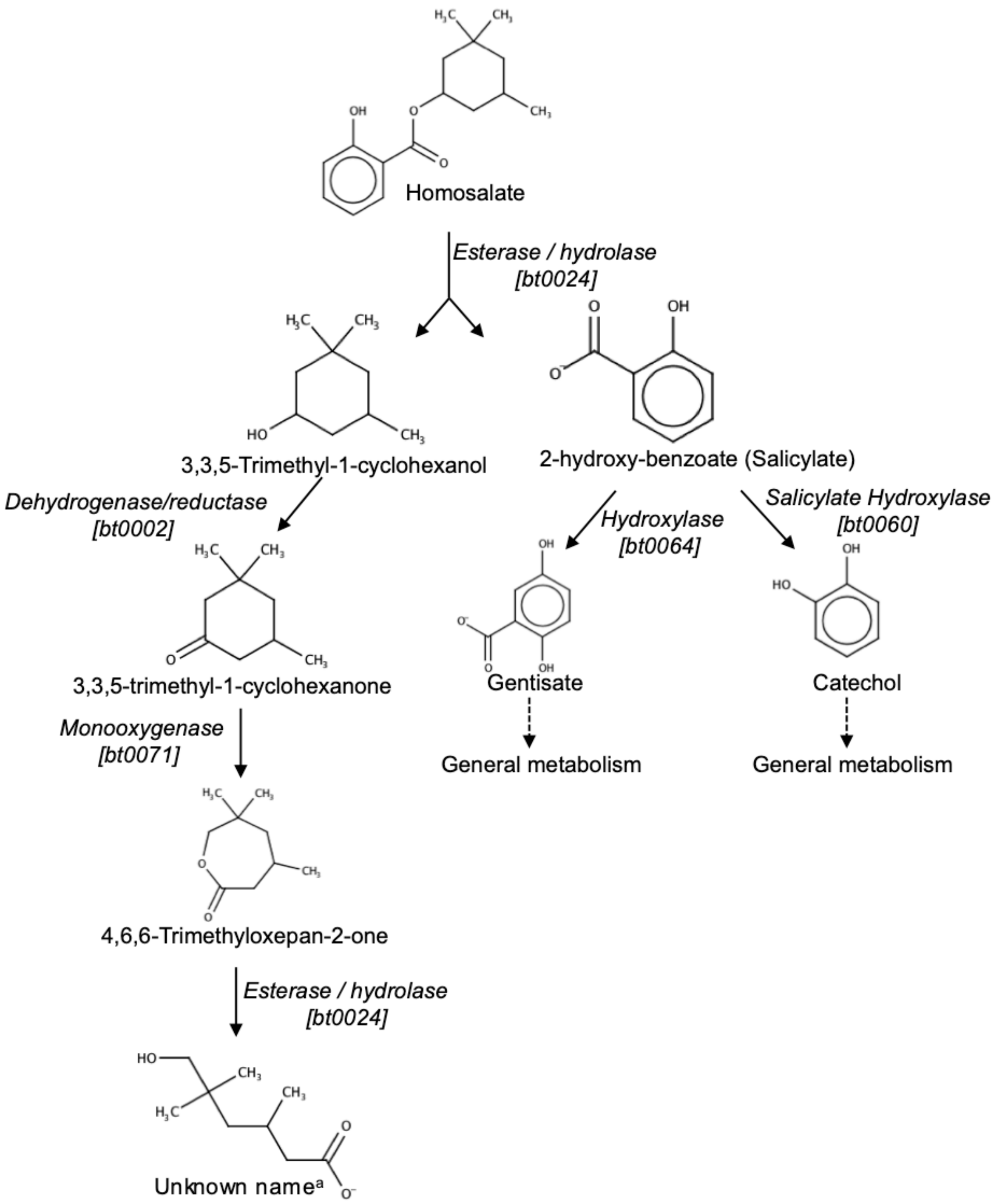

Figure S7. Predicted first steps in a proposed HS degradation pathway. The text between [] refers to the "reaction rule" of the prediction system (http://eawag-bbd.ethz.ch/predict/). [a]=the exact name of this compound could not be determined

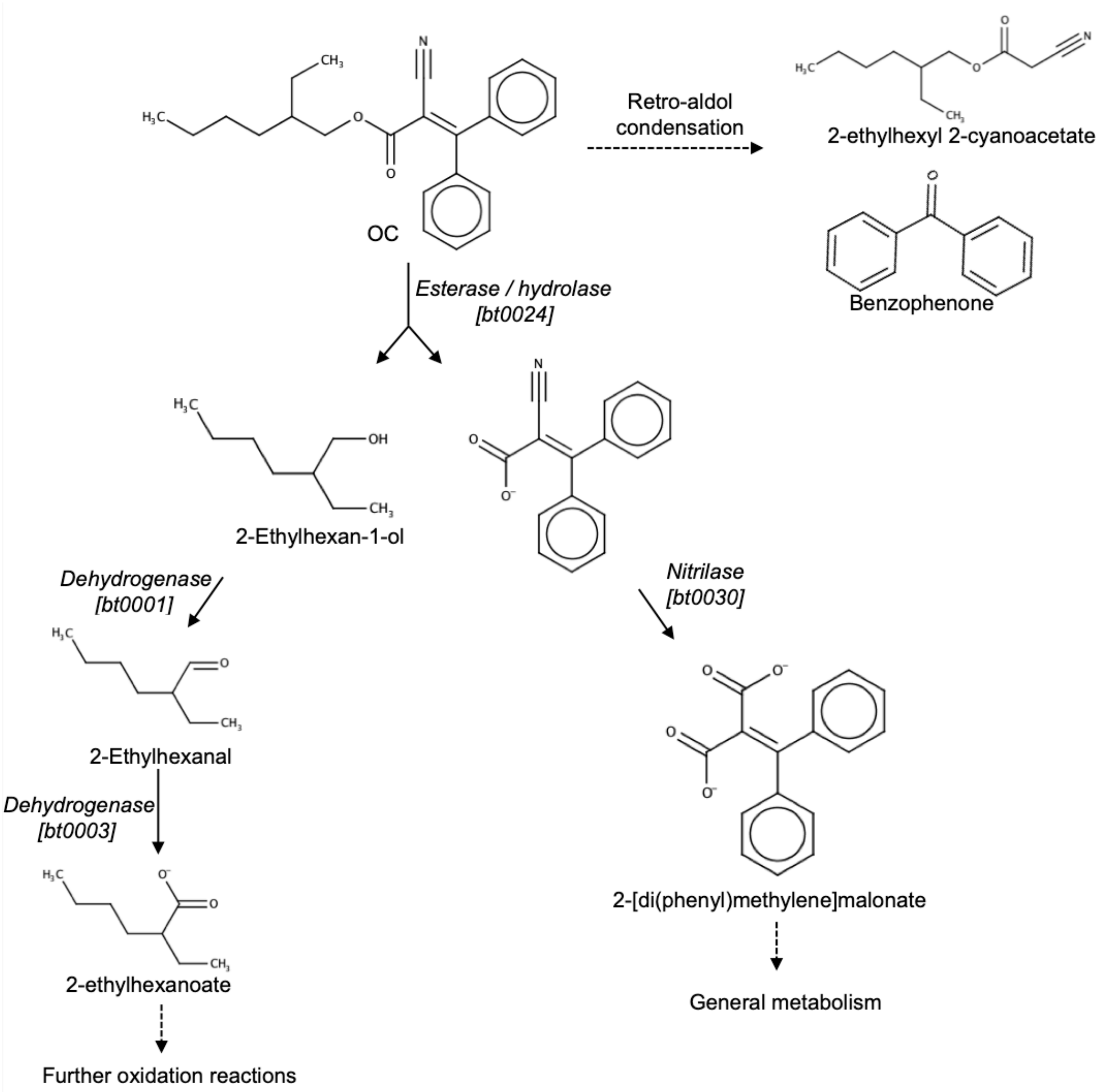

Figure S8. Predicted first steps in a proposed OC degradation pathway. The text between [] refers to the "reaction rule" of the prediction system (http://eawag-bbd.ethz.ch/predict/)
